# Enhancing the Decolorizing and Degradation Ability of Bacterial Consortium Isolated from Textile Effluent Affected Area and Its Application on Seed Germination

**DOI:** 10.1155/2015/628195

**Published:** 2015-01-11

**Authors:** Rashid Mahmood, Faiza Sharif, Sikander Ali, Muhammad Umar Hayyat

**Affiliations:** ^1^Sustainable Development Study Centre, GC University, Lahore 54000, Pakistan; ^2^Institute of Industrial Biotechnology, GC University, Lahore 54000, Pakistan

## Abstract

A bacterial consortium BMP1/SDSC/01 consisting of six isolates was isolated from textile effected soil, sludge, and textile effluent from Hudiara drain near Nishat Mills Limited, Ferozepur Road, Lahore, Pakistan. It was selected because of being capable of degrading and detoxifying red, green, black, and yellow textile dyes. The pH and supplements were optimized to enhance the decolorization ability of the selected consortium. The results indicated that decolorizing ability of consortium for the red, green, black, and yellow dyes was higher as compared to individual strains. The consortium was able to decolorize 84%, 84%, 85%, 85%, and 82% of 200 ppm of red, green, black, yellow, and mixed dyes within 24 h while individual strain required 72 h. On supplementing urea, the consortium decolorized 87, 86, 89, 86, and 83%, respectively, while on supplementing sodium chloride the consortium decolorized 93, 94, 93, 94, and 89% of red, green, black, yellow, and mixed dyes, respectively, which was maximum while in the presence of ascorbic acid and ammonium chloride it showed intermediate results. The effect of untreated and treated dyes was investigated on *Zea mays* L. (maize) and *Sorghum vulgare* Pers. (sorghum). This study will help to promote an efficient biotreatment of textile effluents.

## 1. Introduction

Among the various industrial sectors, the textile and paper industries are especially problematic since they generate significant quantities of wastewater that may have detrimental impacts when released into the environment without any treatment. The environmental problems associated with textile activities are caused mainly by the extensive use of dyes [1]. A great number of these compounds are recalcitrant and have carcinogenic or mutagenic properties. Azo dyes are mostly used in the textile industry due to their extensive variety of color shades and brilliant colors but they are recalcitrant xenobiotics [2, 3]. Ozonation, photooxidation, electrocoagulation, adsorption, activated carbon, froth flotation, reverse osmosis, ion exchange, membrane filtration, and flocculation are applied for color removal from textile effluents [4]. These physicochemical methods are less efficient, have high operational cost, and produce wastes, which are difficult to dispose of. Reduction of color from dye bearing wastewater is a complex problem because of difficulty in treating such wastewaters by conventional treatment methods [5].

Bacteria can be used to remove dyes; some new bacterial strains capable of decolorizing a broad spectrum of dyes have also been isolated and characterized. Though many works have been done on the decolorization of dye by bacteria, work is still required to isolate new bacteria capable of degrading a wide range of structurally different dyes. The study of their physiological characteristics and underlying mechanisms of dye biodegradation at specific temperature and pH is also very important [3, 6, 7]. Various bacterial strains may attack dye molecule at different positions or may use decomposed products produced by another strain for further decomposition [8]. Microbial systems have already been described for remediation of metals and dye contaminated soil and water [9]. The method of microbial degradation of colors involves the reductive cleavage of azo bonds (–N=N–) with the help of azo reductase in anaerobic conditions resulting in the formation of colorless solutions. The effectiveness of microbial decolorization depends on the adaptability and the activity of selected microorganisms [10]. Microbial consortia have been widely used in cleanup of a number of pollutants in laboratory and field bioremediation studies. It is generally thought that microbial consortia are more effective than pure cultures in biodegradation. This is possibly because broader enzymatic capacity is achieved and the formation of toxic intermediate metabolites is counteracted by the selection of these dead end products formed mainly by cometabolism processes. Bacteria express their full capacity to degrade the pollutants in optimal conditions. Temperature, pH, and supplements have great influence on their growth [11, 12].

Phytotoxicity is the most studied parameter amongst the toxicity assays. Genotoxic and cytotoxic effects of textile effluent on the plant root cells were previously demonstrated but some other toxic effects of these effluents on plants are yet to be studied [17]. An assessment of the ecological impact of the environmental pollutants on the plant populations is of great importance as plants are important commercial products and are consumed by humans. Moreover, plants may be used as biosensors of genetic toxicity of the environmental pollutants [13]. There is still room for research to unravel the potential of indigenous microbes for the rehabilitation of the natural resources. Researchers had already worked on the decolorizing ability of bacteria using separate dyes. However, there is a need to treat mixture of dyes as dyes are mostly present in a mixture form in the textile effluent. In this study, isolation and screening of the indigenous bacteria were carried out from textile effluent contaminated sites and a bacterial consortium was developed to reduce the concentration of mixture of dyes. The effect of untreated and treated dyes was investigated on the germination of* Zea mays* L. (Maize) and* Sorghum vulgare* Pers. (Sorghum). This study will help to promote an effective biotreatment of textile effluents.

## 2. Materials and Methods

### 2.1. Sample Collection

Wastewater, sludge, and effected soil samples were collected in screw-capped sterilized bottles for the isolation of bacterial strains from Hudiara drain near Nishat Mills Limited, Ferozepur Road, Lahore 54600, Pakistan One sample of soil, four samples of sludge, and four samples of wastewater were collected from 0, 5, 500, and 1000 meters away from the main outlet [18].

### 2.2. Isolation, Screening, and Identification of the Indigenous Bacteria

The isolation of degrading and detoxifying indigenous bacteria was carried out through serial dilution method on nutrient agar medium at 37°C for 24 h incubation [13, 19]. Isolated bacterial strains were screened out by incubating them on nutrient agar medium containing red (Carmine Red), green (Light Green), black (Eriochrome Black T), and yellow (Metanil Yellow) dyes using 200 ppm of each. The stock cultures of screened bacterial isolates were maintained routinely on the nutrient agar medium and stored at 4°C. The screened bacterial strains were identified based on morphological, biochemical, and physiological properties using the protocol given in Bergey's Manual of Determinative Bacteriology [20].

### 2.3. Development of Bacterial Consortium

The isolates for the consortium development were selected based on criteria: ability to degrade the dyes efficiently (above 60%) and rapidly (within 3 days) and also ability to degrade red, green, black, and yellow dyes. A consortium was developed using combinations of six isolates. A loopful of the selected isolates was individually inoculated for 24 h at 37°C to form a consortium [21].

### 2.4. Determination of Optimal Growth Conditions for Bacterial Consortium

The bacterial isolates were optimized at pH values 6, 6.5, 7, 7.5, 8, and 8.5 and at temperatures 27, 32, 37, 42, and 47°C on nutrient broth having red, green, black, and yellow dyes using 200 ppm of each [22].

### 2.5. Decolorization

Decolorization ability of bacterial isolates and developed consortium was analyzed by using spectrophotometer (SpectroScan 80D UV-VIS) at optimum wavelengths 510 nm for red and black, 410 nm for yellow, 340 nm for green dyes, and 520 nm for mixed dyes. All the decolorization experiments were performed in triplicate on nutrient broth with and without supplements (starch, urea, sodium chloride, ammonium chloride, and ascorbic acid) at 37°C for 24 h incubation. The decolorization activity was expressed in terms of decolorization % using the formula of Cheriaa et al. [14]:

(1) $$\begin{array}{c} \text{Decolorization}\;\%=\frac{At_{0}-At_{f}}{At_{0}}\times 100, \end{array}$$

where *At*_0_ is initial absorbance and *At*_*f*_ is absorbance at incubation time.

### 2.6. Phytotoxicity Studies

In order to assess the toxicity dyes and degraded compound of dye, phytotoxicity tests were performed on* Zea mays* L. CV C1415 (maize) and* Sorghum vulgare* Pers. CV SSG5000 (sorghum). The degraded products of red, green, black, and yellow dyes were extracted and dissolved in 10 mL distilled water for phytotoxicity tests. The phytotoxicity tests were carried out on seeds of maize and sorghum (common agricultural crops of Pakistan). The study was carried out at room temperature (20 seeds of each) by watering 5 mL of red, green, black, and yellow dyes. Control set was carried out using irrigation water at the same time. Germination (%), plumule, and radicle lengths were recorded after 7 days [10].

## 3. Results

### 3.1. Isolation, Screening, and Identification of Dye Decolorizing Bacterial Strains

During isolation process a total of 76 bacterial colonies were observed. From those colonies 21 bacterial strains were isolated as most of the colonies were morphologically similar. The isolates 1, 3, 5, 7, 9, and 20 were screened on basis of the ability to degrade the dyes efficiently more than 60%, within 3 days at 50, 100, 150, and 200 ppm of red, green, yellow, and black dyes. These isolates also exhibited ability to degrade mixture of red, green, black, and yellow dyes. The isolates were identified as* Bacillus subtilis *(Isolate 20),* Bacillus cereus* (Isolate 3),* Bacillus mycoides* (Isolate 1),* Bacillus *sp. (Isolate 5),* Pseudomonas *sp. (Isolate 9), and* Micrococcus *sp. (Isolate 7) by standard physiological, morphological, and biochemical tests using the protocol set in Bergey's Manual of Determinative Bacteriology [20].

### 3.2. Development of Consortium and Decolorization of Textile Dyes

**Figure 1:**
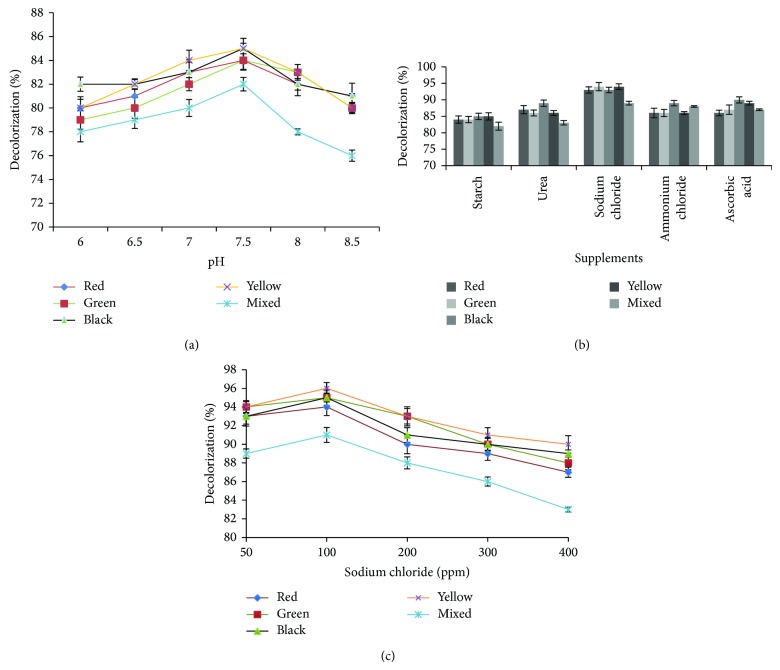
Effect of pH (a), supplements (b), and sodium chloride (c) on decolorizing ability of consortium.

Consortium BMP1/SDSC-01 was developed. It was consisted of* Bacillus subtilis, Bacillus cereus, Bacillus mycoides, Bacillus *sp.,* Micrococcus *sp., and* Pseudomonas *sp. It was observed that isolates alone and in consortium represent an inexpensive tool for the degradation of dyes present in textile effluents. This tool was tested in the present study by examining the decolorization % of red, green, black, and yellow dyes by individual isolates and consortium. It is clearly shown (Tables 1(a) and 1(b)) that pure culture of six isolates decolorized the red dye from 74% to 79% while the consortium decolorized 84%, which is due to accumulative effect of the bacteria present in the consortium. The green dye was decolorized by individual isolate from 75% to 79% and is enhanced by the consortium up to 84%. The black dye was decolorized up to 85% by consortium, while individual strains show maximum efficiency of 79%. Similarly yellow dye decolorization by individual strains was less (74–79%) than the consortium (85%). The consortium performed decolorization of red, green, black, yellow, and mixed dyes at pH 7.5 (incubated at 37°C) up to 84, 84, 85, 85, and 82%, respectively. It was noted that decolorization decreased at pH values 6 and 8.5. The consortium showed maximum decolorization of dyes at temperature 37°C and pH 7.5 (Figure 1).

### 3.3. Enhancement in the Decolorization Ability of Consortium

The consortium was able to decolorize 200 ppm of red (84%), green (84%), black (85%), yellow (85%), and mixed dyes (82%) within 24 h without supplements, whereas on supplementing urea the consortium decolorized 87, 86, 89, 86, and 83%, respectively. On supplementing sodium chloride the consortium decolorized 93, 94, 93, 94, and 89% of red, green, black, yellow, and mixed dyes within 24 h, respectively, which was maximum. Starch supplement did not affect the decolorization of dyes. Ammonium chloride and ascorbic acid also increased the decolorization. The decolorizing efficiency of consortium for red, green, black, and yellow dyes over a range of NaCl concentrations (50 ppm to 400 ppm) was assessed at 37°C. The results indicated that decolorization was maximum at 100 ppm; there was decreasing trend of decolorization at more than 100 ppm NaCl concentrations (Figure 1).

### 3.4. Phytotoxicity Studies

**Figure 2:**
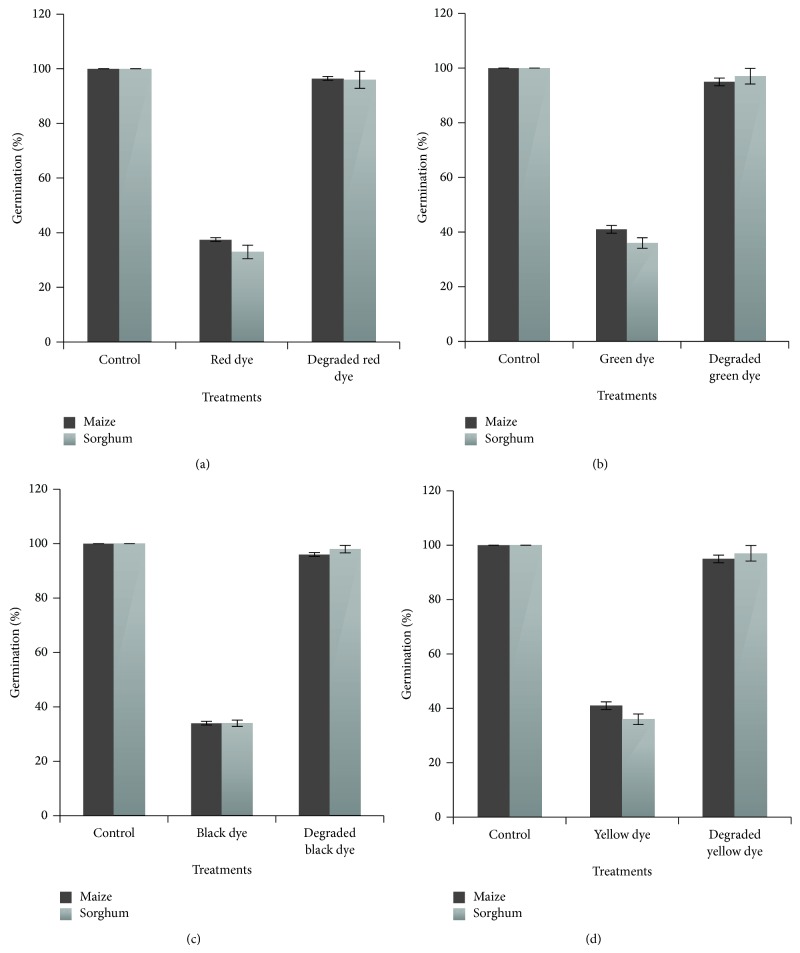
Effects of textile dyes: red (a), green (b), black (c), and yellow (d) before and after the degradation on germination of maize and sorghum.

The water bodies used for irrigation purposes contain untreated effluent from dyeing industry. This practice is of great environmental concern as it associates with biotic and ecosystem health. Soil fertility is directly and indirectly dependent on irrigation water. Biodegradation of effluent leads to generation of various degradation products. Therefore, it is virtually important to study the toxicity impact of these degradation products on plants [23]. The relative sensitivity of* Zea mays* L. and* Sorghum vulgare* Pers. towards the red, green, black, and yellow dyes and its degradation products by consortium were studied. There was 100% germination in control (irrigated water). The germination of* Z. mays* was inhibited to 37.5, 41, 34, and 39%, when treated with 200 ppm of red, green, black, and yellow dyes, respectively. While the germination of* S. vulgare* was also inhibited to 33, 36, 34, and 42% when treated with 200 ppm of red, green, black, and yellow dyes, respectively. The germination of* Z. mays* was 96, 95, 96, and 96% when treated with degradation products of red, green, black, and yellow dyes, respectively. Similarly the germination % of* S. vulgare* was 96, 97, 98, and 97 (Figure 2).

The plumule length and radicle length of* Zea mays* were 20 ± 0.64 and 6 ± 0.96 cm and in case of* Sorghum vulgare *22 ± 1.14 and 6 ± 0.68 cm, respectively. Plumule and radicle length of* Z. mays *weredrastically affected when treated with 200 ppm of red, green, black, and yellow dyes, which was 5 ± 0.08, 6.5 ± 0.95, 4.5 ± 0.48, and 5 ± 0.41 and 2 ± 0.11, 3 ± 0.02, 2 ± 0.042, and 2 ± 0.027, respectively. Plumule and radicle length of* S. vulgare* decreased up to 3 ± 0.22, 4.3 ± 0.81, 3.5 ± 0.38, and 4.1 ± 0.52 and 2 ± 0.024, 2.3 ± 0.11, 2.9 ± 0.12, and 2.4 ± 0.28, respectively, when treated with 200 ppm concentration of red, green, black, and yellow dyes. Plumule and radicle length (cm) of* Z. mays* when treated with degradation products of red, green, black, and yellow dyes were 14, 15, 12, and 13 and 4, 5, 4.5, and 5.2, respectively. Plumule and radicle length (cm) of* S. vulgare* were 7 ± 0.30, 7.5 ± 1.01, 7.5 ± 0.78, and 7.1 ± 0.46 and 4.2 ± 0.15, 4.5 ± 1.04, 49 ± 1.04, and 5.4 ± 1.04, respectively, when treated with degradation products of red, green, black, and yellow dyes (Table 2).

## 4. Discussion

*Bacillus subtilis, Bacillus cereus, Bacillus mycoides, Bacillus *sp.,* Micrococcus *sp., and* Pseudomonas *sp. were isolated from effluent, sludge, and effected soil. A consortium BMP1/SDSC-01 was developed from these isolates. The consortium had ability to degrade red, green, black, and yellow dyes and the mixture of all these dyes. Indigenous isolates have the potential to degrade the dye in natural conditions without high input for their growth. Various strains attack dye molecule at different positions and decomposed products are used by another strain for further decomposition [8]. The main advantage of this consortium is due to its heterogeneity. It is developed from bacteria which were isolated from different sources. They worked together and gave better results for the degradation of dyes. The consortium development in the literature shows that all the researchers developed consortium from single source, whereas Mao et al. [12] reported that heterogeneity of the bacterial community; in this way consortium gave better results in degradation/detoxification of dyes (Table 3). Han et al. [24] also isolated the bacteria for decolorization of textile dyes from the different sources.

The consortium exhibited decolorization of red, green, black, yellow, and mixed dyes at pH 7.5 (incubated at 37°C) up to 84, 84, 85, 85, and 82%, respectively. It is observed that decolorization decreases at pH values 6 and 8.5, as the enzymatic system of bacteria is very sensitive to pH. The consortium exhibited maximum decolorization of dyes at temperature of 37°C (pH 7.5). The consortium was capable of decolorization from 27 to 47°C, but decolorization % decreased above and below 37°C. Moosvi et al. [25] and Jain et al. [11] reported a similar finding showing maximum decolorization at pH 7.5  and 37°C. Presence of different supplements may enhance or retard the decolorizing ability of consortium. When supplements were provided the consortium exhibited a remarkable increase in dye degradation and decolorization. The consortium decolorized 93, 94, 93, 94, and 89% of red, green, black, yellow, and mixed dyes within 24 h, respectively, on supplementation with sodium chloride. These were maximum decolorization percentages shown by the consortium. Starch supplement did not affect the decolorization of dyes. Ammonium chloride and ascorbic acid also increased the decolorization. Similar results were reported by Moosvi et al. [25] and Saratale et al. [10]. This can be applied for decolorization and degradation of effluents at industrial scale.

The biodegradation of effluents by isolated bacteria may lead to generation of a variety of products. Therefore, it is imperative to study the toxicity impact of these degradation products on the life stages of crops so as to overcome yield reduction [10, 13]. Phytotoxicity analysis of textile dyes indicated that germination of plant seeds and radicle and plumule length were affected. In contrast, biodegraded dyes showed good results and toxicity was significantly alleviated. The results indicated that decolorization was maximum at 100 ppm; there was decreasing trend of decolorization at more than 100 ppm NaCl concentrations. It showed linear decrease with increase in concentration; it might be due to role of salt in inhibition of reductase enzyme. Therefore the consortium was competent in decolorization of red, green, black, and yellow dyes between 50 ppm and 400 ppm. The results of present study are in accordance with Wang et al. [15]. Results obtained with red, green, black, and yellow dyes and their degradation products are in agreement with results reported by Saratale et al. [10] and Phugare et al. [17]. For example, seeds of* P. mungo* and* S. vulgare* showed good seed germination in treated water and less plant growth in untreated wastewater [26]. The result indicated that the extracted degradation products by consortium give nontoxic products, resulting in good germination as well as plumule and radicle length of* Z. mays *and* S. vulgare* when compared to dyes. Toxicity of textile dyes is not restricted to plants only but the adverse effects of these have been reported [27] to mammals also. Exposure to these toxic textile dyes present in effluents and sludge can cause chromosomal alteration, DNA mutation, abnormal sperm, foetus loss, birth defects, and altered sex ratio in mammals. The treatment by bacterial consortium can be used effectively in reducing these adverse effects. As phytotoxicity studies revealed that the biodegradation of the red, green, black, and yellow dyes by the consortium led to detoxification of the pollutant, the bacterial consortium can be the best tool for the biotreatment of textile effluent.

## 5. Conclusion

A bacterial consortium BMP1/SDSC/01 consisting of six bacterial isolates (*Bacillus cereus, Bacillus mycoides, Bacillus subtilis, Bacillus *sp.,* Micrococcus *sp., and* Pseudomonas *sp.) belonging to three genera exhibited good decolorization, degradation, and detoxification of red, green, black, and yellow dyes and mixture of all these dyes. The consortium BMP1/SDSC/01 has not been reported so far to our knowledge for dyes decolorization and degradation. The use of urea, sodium chloride, ammonium chloride, and ascorbic acid enhances the decolorization ability of consortium BMP1/SDSC/01. The water bodies used for irrigation purposes contain untreated effluent from dyeing industry. Thus, phytotoxicity studies on* Z. mays *and* S. vulgare* revealed that the biodegradation of the red, green, black, and yellow dyes by the consortium BMP1/SDSC/01 led to detoxification of the pollutant. Bioremediation gives an advantage for treatment of textile effluents and studies regarding the potential of consortium at large scale are in progress.

## Figures and Tables

**(a):** 

Sr. number	Dyes	Chemical class	λ_max_ nm	Decolonization (%)						
				Incubation time						
				72 h						24 h
				*Bacillus mycoides *	*Bacillus cereus *	*Bacillus * sp.	*Pseudomonas *sp.	*Micrococcus *sp.	*Bacillus subtilis *	Consortium
1	Red	Carmine acid	510	74	76	75	79	78	77	84
2	Green	Benzenesulfonate	340	76	75	79	76	79	78	84
3	Black	Monoazo	510	73	74	74	77	77	79	85
4	Yellow	Monoazo	410	76	74	77	78	79	79	85

**(b):** 

Sr. number	Dyes	Formal name	Chemical structure
1	Red	Carmine Red	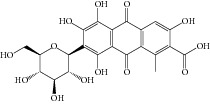

2	Green	Light Green	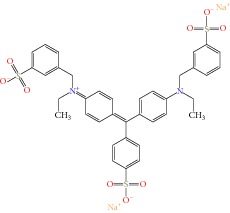

3	Black	Eriochrome Black T	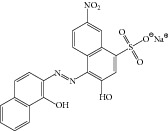

4	Yellow	Metanil Yellow	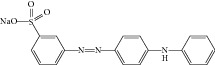

**Table 2:** Phytotoxicity test of different treated and untreated textile dyes.

Treatments	Maize		Sorghum	
	Plumule (cm)	Radicle (cm)	Plumule (cm)	Radicle (cm)
Control (irrigation water)	20 ± 0.64	6 ± 0.96	22 ± 1.14	6 ± 0.68
Red dye	5 ± 0.08	2 ± 0.11	3 ± 0.22	2 ± 0.024
Degraded red dye	14 ± 0.79	4 ± 0.04	7 ± 0.30	4.2 ± 0.15
Green dye	6.5 ± 0.59	3 ± 0.02	4.3 ± 0.81	2.3 ± 0.11
Degraded green dye	15 ± 1.3	5 ± 0.31	7.5 ± 1.01	5.4 ± 1.04
Black dye	4.5 ± 0.48	2 ± 0.040	3.5 ± 0.38	2.9 ± 0.12
Degraded black dye	12.5 ± 1.40	4.5 ± 0.63	7.5 ± 0.79	4.9 ± 1.04
Yellow dye	5 ± 0.41	2 ± 0.027	4.1 ± 0.52	2.4 ± 0.28
Degraded yellow dye	13 ± 0.72	5.2 ± 0.14	7.1 ± 0.46	5.4 ± 1.05

**Table 3:** Decolorization rate of different bacterial consortium.

Sr. number	Consortium/bacteria	Decolorization rate (mg/h)	Name of dye	Reported by
1	*Citrobacter* sp. CK3	5.55	Reactive Red 180	Wang et al. [3]

2	Consortium GR (*Proteus vulgaris* and*Micrococcus glutamicus*)	1.60	Azo dye Scarlet R	Saratale et al. [10]

3	Consortium DAS (3 strains of *Pseudomonas *sp.)	0.43	Reactive Orange 16	Jadhav et al. [13]

4	Consortium CM-4 (*Agrobacterium radiobacter, Aeromonas hydrophila, Bacillus *sp., and* Sphingomonas paucimobilis*)	2.08	Crystal violet	Cheriaa et al. [14]

5	Consortium CM-4 (*Agrobacterium radiobacter, Aeromonas hydrophila, Bacillus *sp., and* Sphingomonas paucimobilis*)	2.08	Malachite Green	Cheriaa et al. [14]

6	*Bacillus *sp. YZU1	0.83	Reactive Black 5	Wang et al. [15]

7	*Brevibacillus laterosporus* MTCC	0.91	Golden Yellow HER	Gomare et al. [16]

8	Consortium BMP1/SDSC/01 (*Bacillus mycoides, Bacillus cereus, Bacillus *sp., *Pseudomonas* sp., *Micrococcus* sp., and *Bacillus subtilis*)	7.00	Red	Present Study

	Consortium BMP1/SDSC/01 (*Bacillus mycoides, Bacillus cereus, Bacillus *sp., *Pseudomonas* sp., *Micrococcus* sp., and *Bacillus subtilis*)	7.00	Green	Present Study

	Consortium BMP1/SDSC/01 (*Bacillus mycoides, Bacillus cereus, Bacillus *sp., *Pseudomonas* sp., *Micrococcus* sp., and *Bacillus subtilis*)	7.08	Black	Present Study

	Consortium BMP1/SDSC/01 (*Bacillus mycoides, Bacillus cereus, Bacillus *sp., *Pseudomonas* sp., *Micrococcus* sp., and *Bacillus subtilis*)	7.08	Yellow	Present Study
